# Controlled division of cell-sized vesicles by low densities of membrane-bound proteins

**DOI:** 10.1038/s41467-020-14696-0

**Published:** 2020-02-14

**Authors:** Jan Steinkühler, Roland L. Knorr, Ziliang Zhao, Tripta Bhatia, Solveig M. Bartelt, Seraphine Wegner, Rumiana Dimova, Reinhard Lipowsky

**Affiliations:** 1 Max Planck Institute of Colloids and Interfaces, Science Park Golm, 14424 Potsdam, Germany; 2 Max Planck Institute for Polymer Research, Ackermannweg 10, 55128 Mainz, Germany

**Keywords:** Membrane biophysics, Synthetic biology, Biophysical chemistry, Biological physics

## Abstract

The proliferation of life on earth is based on the ability of single cells to divide into two daughter cells. During cell division, the plasma membrane undergoes a series of morphological transformations which ultimately lead to membrane fission. Here, we show that analogous remodeling processes can be induced by low densities of proteins bound to the membranes of cell-sized lipid vesicles. Using His-tagged fluorescent proteins, we are able to precisely control the spontaneous curvature of the vesicle membranes. By fine-tuning this curvature, we obtain dumbbell-shaped vesicles with closed membrane necks as well as neck fission and complete vesicle division. Our results demonstrate that the spontaneous curvature generates constriction forces around the membrane necks and that these forces can easily cover the force range found in vivo. Our approach involves only one species of membrane-bound proteins at low densities, thereby providing a simple and extendible module for bottom-up synthetic biology.

## Abstract (web summary)

Membrane fission of a cell into two daughters is a core ability of cell-based life. Here the authors show that in artificial cells division can be controlled by regulating membrane curvature using low protein density.

## Introduction

Living cells have the amazing ability to divide into several daughter cells. This ability provides the basis for the population growth of unicellular organisms and for the individual development of higher organisms. All present-day cells are enclosed by their plasma membranes, which represent fluid bilayers of lipids and proteins. The division of such a cell necessarily involves the deformation and fission of its plasma membrane, which proceeds via a sequence of well-defined membrane shapes. If we start from a quasi-spherical cell, the (apparent) volume-to-area ratio must first decrease, leading to shapes of lower symmetry, such as prolates, which eventually transform into dumbbells. The latter shapes consist of two subcompartments connected by a narrow or closed membrane neck. Finally, the membrane neck undergoes fission and the cell divides into two daughter cells. The membrane processes just described can also be observed for model membranes, such as giant unilamellar vesicles (GUVs), which often attain dumbbell shapes. Furthermore, several methods have been proposed to induce the fission of the GUV membranes, for example, by the reconstitution of bacterial FtsZ filaments^1^, by protein crowding^2^, and by the mechanical splitting of giant vesicles at microfluidic junctions^3^.

Here, we introduce a powerful approach that allows us to divide giant vesicles in a highly controlled and reliable manner. Our approach is based on His-tagged fluorescent proteins bound to the GUV membranes. As a specific example, we use green fluorescent proteins (GFPs). We explore the dilute regime, in which the membrane-bound proteins are well separated from each other, thereby avoiding the complications of protein crowding. The proteins are added to the exterior solution of the GUVs, which leads to asymmetric GUV membranes with a certain preferred or spontaneous curvature. By calibrating the fluorescence of the membrane-bound proteins, the protein coverage of the membranes is shown to increase linearly with the protein solution concentration over a wide concentration range. A detailed comparison between theory and experiment also reveals a linear relationship between the GFP coverage and the spontaneous curvature. As a consequence, we are able to control the spontaneous curvature and thus the vesicle shape in a systematic and quantitative manner. Increasing the spontaneous curvature, we observe the formation of dumbbell shapes and the subsequent fission of membrane necks. The GFP-induced fission is driven by constriction forces that are generated by the spontaneous curvature and are comparable in magnitude to those generated by protein complexes^4–6^ in vivo. In this way, we reveal a simple and robust curvature-elastic mechanism for vesicle division. This mechanism does not depend on the precise nature of the molecular interactions that generate the spontaneous curvature of the GUV membranes as we demonstrate at the end by using His-tagged iLid proteins rather than His-tagged GFP.

## Results

### GFP Coverage and spontaneous curvature

GUVs were prepared from ternary lipid mixtures of POPC, POPG, and cholesterol. The GUVs were exposed to GFP dissolved in the exterior solution. The GFPs had a His-tag, by which they could bind in a reversible manner to anchor-lipids within the outer leaflets of the GUV membranes. These anchor-lipids were provided by 0.1 or 1 mol% DGS-NTA. The resulting GFP coverage Γ on the outer leaflet of the vesicle membranes was estimated by calibrating the fluorescence signal of GFP with the corresponding signal of another lipid-dye similar to GFP, see “Methods” section for technical details. The GFP coverage Γ, which represents the number of lipid-anchored GFP molecules per membrane area, was found to increase linearly with the GFP solution concentration *X* (Supplementary Fig. 1). For all concentrations, the average separation of the lipid-anchored GFPs exceeded 24 nm, which is much larger than the GFP’s lateral size of about 3 nm^7^. As a consequence, the whole concentration range explored here belongs to the dilute regime in which we can ignore steric interactions between the membrane-bound molecules.

**Fig. 1:**
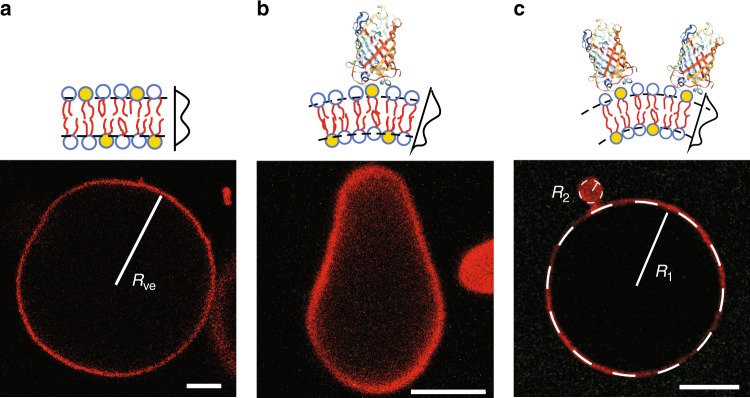
Curvature of GUV membranes induced by His-tagged GFP. Cartoons of lipid bilayers and confocal images of GUVs **a** in the absence of GFP, that is, for GFP solution concentration *X* = 0 nM and GFP coverage Γ = 0 μm^−1^. **b** For *X* = 0.78 nM and Γ = 5.4 μm^−2^. **c** For *X* = 7.8 nM and Γ = 54 μm^−2^. In the cartoons, the anchor-lipids (yellow) bind the His-tags of the bulky GFP barrels (multi-colored). These protein barrels have an extension that is comparable to the lipid bilayer thickness of about 4 nm. The average separation of the membrane-bound GFPs is much larger than the bilayer thickness and equal to 136 nm for the right-most cartoon with Γ = 54 μm^−2^ (not drawn to scale). In the confocal images, the GUV membranes (red) were doped with 0.1 mol% lipid dye. **a** The image displays a spherical GUV with vesicle size *R*_ve_, related to the membrane area *A* via ${R}_{{\rm{ve}}}\equiv \sqrt{A/(4\pi )}=14.5$ μm. **c** The image shows a dumbbell shape consisting of two spherical membrane segments with radii *R*_1_ = 7.27 μm and *R*_2_ = 0.92 μm. These two segments are connected by a closed membrane neck with neck curvature ${M}_{{\rm{ne}}}=\frac{1}{2}({R}_{1}^{-1}+{R}_{2}^{-1})=0.61\,$ μm^−1^ (see the “Methods” section, Eq. (4)). All scale bars: 5 μm.

**Fig. 2:**
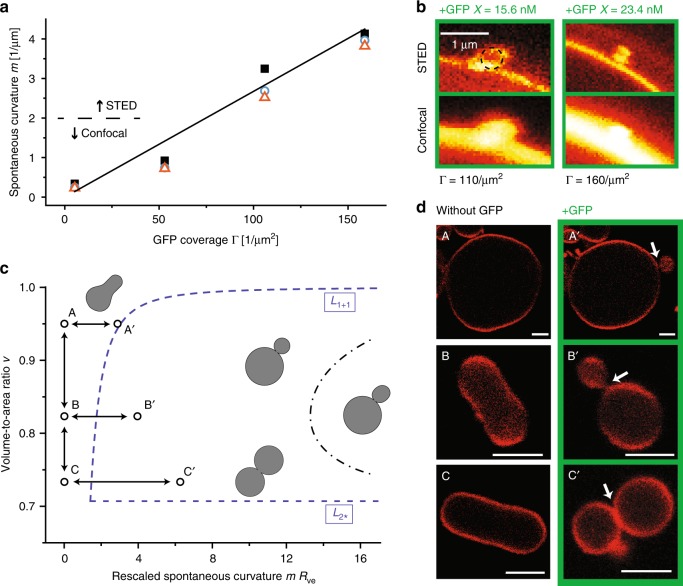
Spontaneous curvature and morphology diagram. **a** Spontaneous curvature *m* generated by GFP coverage Γ: We studied four vesicle populations with four different GFP coverages as obtained for 0.1 mol% anchor-lipids and molar GFP concentrations *X* = 0.78, 7.8, 15.6, and 23.4 nM. For the two lower concentrations *X* = 0.78 and 7.8 nM, the dummbell-shaped vesicles were imaged by confocal microscopy, whereas we used STED microscopy to resolve the small buds formed for the higher concentrations *X* = 15.6 and 23.4 nM. For each vesicle population, we selected the three vesicles with the largest neck curvature *M*_ne_ as defined in Eq. (5) from at least three independent experiments. These three curvature values provide the three data points (black squares, red triangles, green circles) included for each Γ-value. The straight line represents the least-squares fit through the black squares and has a slope of about 27 nm ($R^{2}_{\mathrm{adj}} \simeq 0.98$). **b** Confocal and STED images of small buds as obtained for GFP concentrations *X* = 15.6 and 23.4 nM. For the analysis of the STED images, see Supplementary Fig. 2. **c** Morphology diagram as a function of two dimensionless shape parameters, the rescaled spontaneous curvature *mR*_ve_ and volume-to-area ratio *v*. Dumbbell-shaped vesicles with a closed membrane neck are found between the two dashed lines *L*_1+1_ and *L*_2*_ (blue). The latter subregion contains the dash-dotted line, at which the spherical buds become unstable and transform into prolate buds. The shapes denoted by A, B, and C correspond to three GUVs in the absence of GFP. Adding GFP to the exterior solution, the three GUVs transformed into the dumbbell shapes A${}^{\prime}$, B${}^{\prime}$, and C${}^{\prime}$. **d** Confocal images of the six vesicle shapes A, B, and C (left column) as well as A${}^{\prime}$, B${}^{\prime}$, and C${}^{\prime}$ (right column). The white arrows indicate the positions of the closed membrane necks. The corresponding parameter values are displayed in Table 1. All scale bars: 5 μm. Source data for panels **a** and **c** are provided in the Source Data file.

As we increased the GFP concentration *X* and, thus, the GFP coverage Γ of the outer membrane leaflet, the two sides of the membranes became more and more asymmetric, generating a preferred or spontaneous curvature that increased with Γ. The membranes then tried to adapt to this spontaneous curvature by bulging towards the GFPs, thereby forming more highly curved segments as illustrated in Fig. 1. To determine the relationship between the GFP coverage Γ and the spontaneous curvature *m* in a quantitative manner, we first focussed on those GUVs with dumbbell shapes, consisting of two spherical membrane segments connected by a closed membrane neck. The neck is stably closed as long as the neck curvature *M*_ne_, which depends on the curvature radii of the two spherical segments (see the “Methods” section, Eq. (4) and Fig. 1c) does not exceed the spontaneous curvature. Thus, in order to obtain reliable estimates for the spontaneous curvature, we selected those closed necks that had the largest neck curvature *M*_ne_ for a given value of the GFP concentration *X*. These neck curvatures provide an estimate for the spontaneous curvature *m* generated by the lipid-anchored GFP (see Fig. 2a). When the GFP coverage Γ exceeded about 75 μm^−2^, the curvature radius of the smaller sphere could no longer be resolved by confocal microscopy and we used stimulated emission depletion (STED) microscopy to determine this radius (Fig. 2b and Supplementary Fig. 2). The data in the latter figure show that this curvature increases linearly with the GFP coverage Γ and the GFP solution concentration *X* according to

1 $$m=\Gamma\; \times\, 27\ {\rm{nm}}=\frac{\alpha}{\upmu{\mathrm{m}}}\ \frac{X}{{\rm{nM}}}$$

with the prefactor *α* = 0.186 for 0.1 mol% and *α* = 1.86 for 1 mol% anchor-lipids. Equation (1) implies that the GFP-generated spontaneous curvature *m* is quite large and comparable in size to the spontaneous curvature estimated for membrane-bound amphiphysin^8^ based on tube-pulling experiments. Indeed, in the dilute regime with Γ < 1000/μm^2^, three different estimates for the spontaneous curvature have been obtained in ref. ^8^, which correspond to *m* = Γ*L*_*m*_ with the length scale *L*_*m*_ given by 10, 25, and 50 nm, respectively. Therefore, the spontaneous curvature generated by lipid-anchored GFP is certainly comparable in size to the one generated by membrane-bound amphiphysin and may even be three times larger.

### Morphology diagram of GUVs

To corroborate Eq. (1), we systematically studied the GUV morphologies as a function of spontaneous curvature *m* and volume-to-area ratio *v* (see “Methods” section, Eq. (6)). The spontaneous curvature was controlled via the GFP solution concentration *X* in the exterior solution whereas the volume-to-area ratio *v* was varied via the osmotic conditions. To avoid mechanical perturbations of the GUVs by hydrodynamic flows, we used diffusional exchange of GFP and osmolytes into  microfluidic dead-end channels^9^, see “Methods” section. The resulting morphology diagram is shown in Fig. 2c. We started from GUVs exposed to symmetric buffer conditions containing small solutes but no GFP, corresponding to vanishing spontaneous curvature. We then varied the volume of the GUVs by changing the small solute concentration and the spontaneous curvature by adding GFP to the exterior solution. Three examples for GUVs observed for symmetric buffer conditions in the absence of GFP are provided by the shapes A, B, and C in the left column of Fig. 2d. When we exposed these GUVs to sufficiently large GFP concentrations, they transformed into the dumbbell shapes A${}^{\prime}$, B${}^{\prime}$, and C${}^{\prime}$ with a closed membrane neck as displayed in the right column of Fig. 2d. The three GUVs labeled by A, B, and C had different sizes and volume-to-area ratios (see Table 1). When we decreased the spontaneous curvature by decreasing the GFP concentration, the closed membrane necks opened up (Movie 1), directly demonstrating the reversible binding between the His-tagged GFPs and the NTA anchor-lipids. Compared to previous estimates^10^, we observed relatively fast GFP dissociation (Supplementary Figs. 3 and 4), indicating that the GFP–NTA binding was predominantly mediated by monovalent interactions. Furthermore, using photon counting statistics^11^ (see Supplementary Fig. 5 and Supplementary Methods), we obtained direct evidence that the used membrane-bound GFP mutant remained monomeric and did not dimerize, in accordance with previous experimental results^12^.

**Table 1:** Vesicle size *R*_ve_, volume-to-area ratio *v*, molar GFP concentration *X*, mol% DGS-NTA anchor-lipids, GFP coverage Γ, spontaneous curvature *m*, and rescaled spontaneous curvature *mR*_ve_ for the six vesicle shapes A, A${}^{\prime}$, B, B${}^{\prime}$, C, and C${}^{\prime}$ depicted in Fig. 2c, d.

Shape	A	A${}^{\prime}$	B	B${}^{\prime}$	C	C${}^{\prime}$	D^†^	E^†^	F^†^
*R*_ve_ [μm]	15.1	15.1	2.7	2.7	4.2	4.2	6.16	4.60	4.10
*v*	0.95	0.95	0.83	0.83	0.73	0.73	0.93	0.70	0.71
*X* [nM]	0	0.78	0	7.8	0	7.8	31.2	39	15.6
NTA [mol%]	0.1	0.1	0.1	0.1	0.1	0.1	0.1	0.1	1
Γ [μm^−2^]	0	5.38	0	53.8	0	53.8	216	269	1076
*m* [μm^−1^]	0	0.145	0	1.45	0	1.45	5.81	7.26	29
*mR*_ve_	0	2.19	0	3.9	0	6.1	36.0	33.4	119
*M*_ne_ [μm^−1^]		0.212		0.204		0.340	0.441	0.308	0.345
*m* − *M*_ne_ [μm^−1^]		<0		1.246		1.11	5.37	6.95	28.7
*f* [pN]		<0		6.1		5.5	26.6	36.4	142

**Fig. 3:**
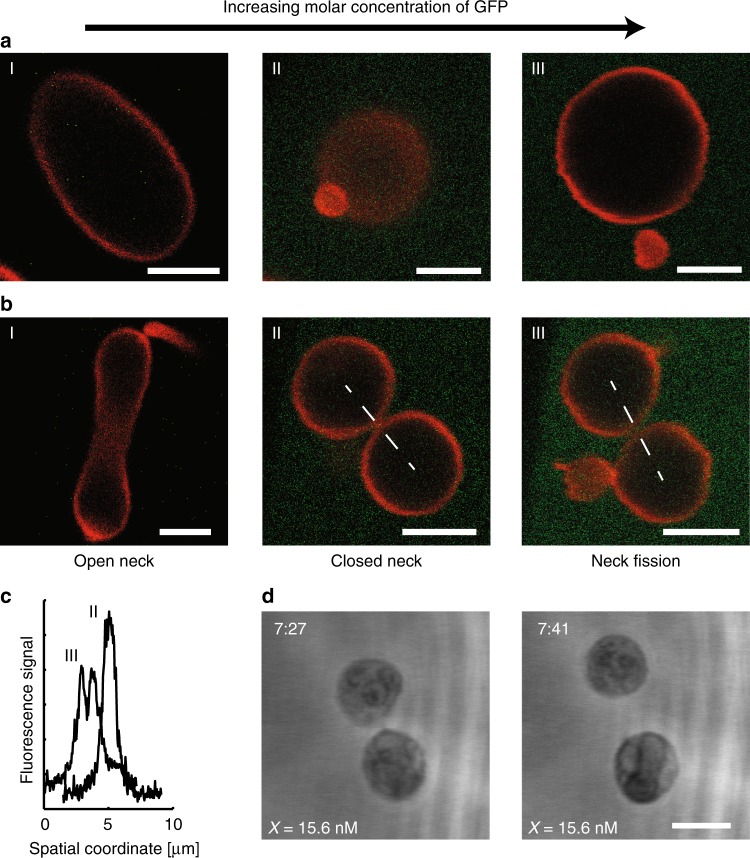
Division of GUVs (red) by increasing the GFP concentration. **a** Asymmetric division of a single GUV into one large and one small daughter vesicle. **b** Symmetric division of a  single GUV into two daughter vesicles of equal size. The division process starts, in the absence of GFP, from a certain vesicle shape as displayed in I. Addition of GFP then transforms each GUV into two spherical membrane segments that are connected by a closed membrane neck as in II. A further increase in the GFP concentration leads to the cleavage of the neck and to the division of the GUV as shown in III. The GUVs in (**a**,III) and (**b**,III) are denoted by D^†^ and E^†^, respectively. The key parameter that controls the relative size of the two daughter vesicles is the volume-to-area ratio *v* (see the “Methods” section, Eq. (6)). **c** Profile of lipid fluorescence along the white dashed line in (**b**,II) indicates a closed neck but neck fission along the white dashed line in (**b**,III). **d** Two snapshots from Supplementary Movie 2: The GUV had the dumbbell shape F^†^ when it underwent neck fission after about 7:27 min:s, resulting in two daughter vesicles that diffused freely away from each other and were completely separated at 7:41 min:s. The parameters for the shapes D^†^, E^†^, and F^†^ are included in Table 1. The membranes in **a** and **b** contained 0.1 mol%, the one in **d** 1 mol% anchor-lipids. All scale bars: 5 μm. Source data for panel **c** are provided in the Source Data file.

The last three rows display the neck curvature *M*_ne_ (see “Methods” section, Eq. (5)), the curvature difference *m* − *M*_ne_, and the constriction force *f*, see Eq. (2), for the dumbbell shapes A${}^{\prime}$, B${}^{\prime}$, and C${}^{\prime}$. The negative value of *f* for A${}^{\prime}$ implies that the membrane neck is slightly open, see location of A${}^{\prime}$ in Fig. 2c. The dumbbell shapes D^†^, E^†^, and F^†^ in the last three columns underwent neck fission (Fig. 3).

Our experimentally observed GUV morphologies, which are summarized in Fig. 2c, d and Table 1, are in very good agreement with theoretical results obtained from the spontaneous curvature model^13,14^. According to this model, dumbbell shapes with a closed membrane neck are obtained within a certain subregion of the morphology diagram. This subregion is bounded by the lines *L*_1+1_ and *L*_2*_ corresponding to the two dashed lines in Fig. 2c (see “Methods“ section for technical details). For all points on the horizontal line *L*_2*_, the vesicle attains the same limit shape consisting of two equally sized spheres with radius ${R}_{{\rm{ve}}}/\sqrt{2}$. The latter shapes are of particular interest if one wants to achieve the division of GUVs into two daughter vesicles of equal size.

### Fission of membrane necks induced by spontaneous curvature

The preceding discussion of the morphology diagram focussed on the stability of membrane necks against neck opening. In addition, we implicitly assumed that the closed membrane necks were stable against fission. The latter assumption was indeed valid for the range of spontaneous curvature values displayed in Fig. 2a and explored in Fig. 2b–d. However, when we exposed dumbbell-shaped GUVs with closed membrane necks to sufficiently large GFP concentrations (Supplementary Fig. 6), which generated sufficiently large spontaneous curvatures, we started to observe neck fission and GUV division. Three examples for such a fission and division process are shown in Fig. 3. No residual connections remained between the newly formed daughter vesicles, as confirmed by the absence of lipid diffusion between the vesicles (Supplementary Fig. 7).

**Fig. 4:**
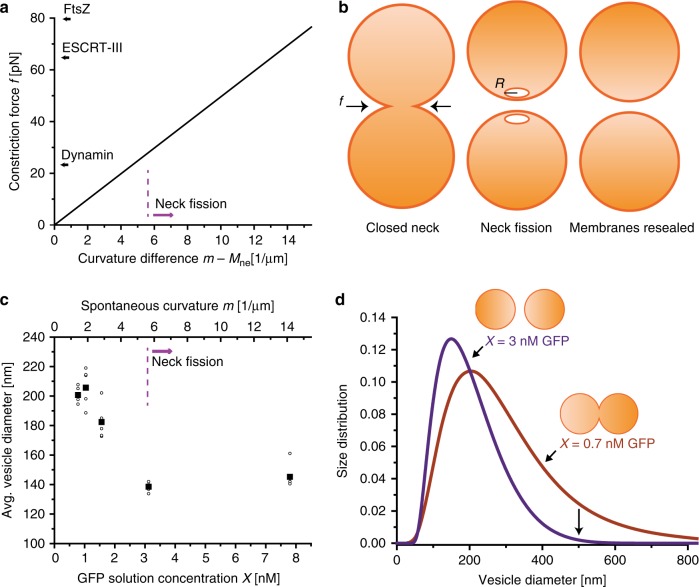
Vesicle division arising from constriction forces generated by spontaneous curvature. **a** Constriction force *f* compressing the closed membrane neck as a function of the curvature difference *m − M*_ne_. The straight line corresponds to Eq. (2) with *κ* = 48*k*_B_*T*. For comparison, the plot also includes literature values for the constriction forces as generated by the specialized protein complexes of dynamin^4^, ESCRT-III^5^, and FtsZ^6^. **b** Neck fission and division of a symmetric dumbbell with a closed membrane neck on the left to the two-vesicle state on the right. The free energy barrier between these two states is provided by the intermediate state with two daughter vesicles, each of which has a small membrane pore arising from the broken neck. The radius *R* of the pores is determined by the neck size before fission. **c** Average vesicle diameter of LUVs measured by dynamic light scattering as a function of GFP concentration *X*. Error bars indicate standard deviations from six measurements on the same sample. In total, two independent repeat experiments were performed. The upper *x*-axis with the spontaneous curvature *m* is obtained from the calibration curve in Supplementary Fig. 1 and the data in Fig. 2a, as described by Eq. (1). **d** Size distribution of LUVs obtained for GFP concentration *X* = 0.7 and 3 nM by dynamic light scattering. These two *X*-values generate a spontaneous curvature of about 1.5 and 5.5 μm^−1^. The data in **c** and **d** were obtained for 1 mol% anchor-lipids. Source data for panels **a**, **c**, and **d** are provided in the Source Data file.

These experimental observations confirm the recent theoretical prediction that neck fission can be achieved by simply increasing the membrane’s spontaneous curvature.^14^ As explained in the “Methods” section, the spontaneous curvature *m* is predicted to generate the constriction force

2 $$f=8\pi \kappa \left(m-{M}_{{\rm{ne}}}\right)$$

around the membrane neck which is proportional to the curvature difference *m − M*_ne_ and to the bending rigidity *κ*. For the lipid membranes used here, the bending rigidity had the value *κ* = 48*k*_B_*T* (see the “Methods” section). The resulting constriction force *f* is depicted in Fig. 4a. For the dividing GUVs, the constriction force *f* as given by Eq. (2) can be well approximated by *f* ≈ 8*πκm* which is independent of the GUV geometry.

Inspection of Fig. 4a reveals that the whole range of constriction forces as generated by protein complexes in vivo can be obtained by increasing the curvature difference *m* − *M*_ne_ up to about 16 μm^−1^, corresponding to a GFP coverage of about 600 μm^−2^. The membrane-anchored GFP molecules then have an average separation of about 41 nm, which implies that the observed fission processes were not related to protein crowding or polymerization. In previous studies^2,15^, the crowding regime of His-tagged GFP has been investigated using GUV membranes with 20 mol% NTA anchor-lipids. Furthermore, in ref. ^2^, the GUV membranes were exposed to a GFP solution concentration of 5 and 20 μM. Thus, in the latter study, the anchor-lipid mole fraction was at least 20 times higher and the GFP solution concentration was at least 128 times larger than in our systems. It is important to note that we could also exclude significant enrichment or depletion of GFP in the neck region (Supplementary Fig. 5).

The process of symmetric division into two daughter vesicles of equal size is schematically shown in Fig. 4b. This process represents a topological transformation from the one-vesicle state on the left to the two-vesicle state on the right and involves the Gaussian curvature modulus^16^. As explained in “Methods“ section, the process is strongly exergonic but has to overcome a free energy barrier associated with the formation of two small membrane pores replacing the membrane neck (Fig. 4b). Interestingly, neck fission was also observed for GUVs with complex, non-axisymmetric shapes (Supplementary Fig. 8).

To improve the statistics of our measurements, we also studied large populations of smaller vesicles, conventionally called large unilamellar vesicles (LUVs), that had diameters below optical resolution. The lipid bilayers of these vesicles contained 1 mol% anchor-lipids. The smaller vesicles were incubated with increasing GFP concentrations and their size was determined by dynamic light scattering. The vesicles were again deflated osmotically to reduce their volume and to create sufficient excess area for division. When we increased the GFP concentration to about 2.7 nM, corresponding to a spontaneous curvature of about 5 μm^−1^, the average diameter of the vesicles decreased from about 200  to about 140 nm (Fig. 4c). Likewise, the size distribution of the vesicles as obtained by dynamic light scattering was shifted towards smaller sizes as we increased the GFP concentration (Fig. 4d). These independent experiments on LUVs are completely consistent with our results for GUVs. Thus, we conclude that the LUVs were also divided by osmotically driven budding and neck closure, followed by neck fission induced by a further increase in spontaneous curvature.

### Division of GUVs coupled to reservoirs for membrane area

Both the division of GUVs and the division of living cells requires the formation of two membrane sub-compartments which is only possible by decreasing the volume-to-area ratio *v*. In the experiments described so far, the decreased *v*-values were obtained by reducing the vesicle volume via osmotic deflation. In contrast, cells typically maintain fixed osmotic conditions and are thought to increase the area of their plasma membranes by retrieving area from membrane reservoirs. In the context of GUVs, such area reservoirs can be created in the form of membrane nanotubes, which are induced by large spontaneous curvatures and can be retracted into the mother vesicles, thereby increasing the robustness of these vesicles against mechanical perturbations^17^.

**Fig. 5:**
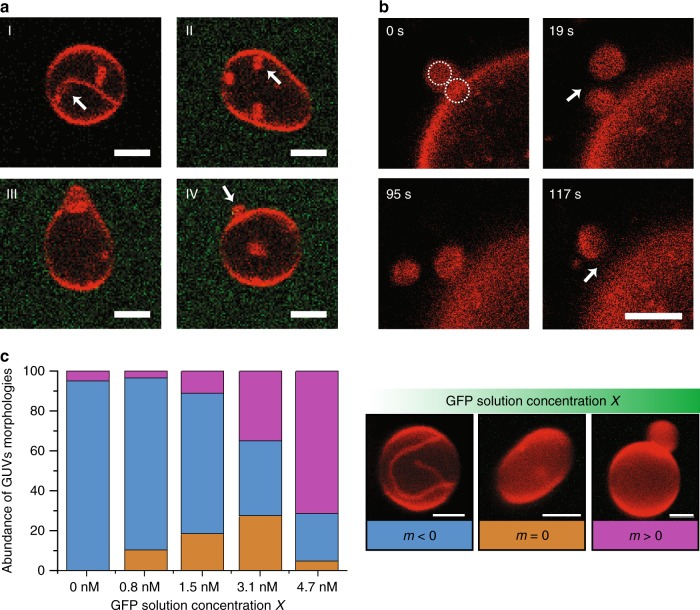
Behavior of GUVs with area reservoirs provided by nanotubes. **a** Series of (transient) shapes observed for a single GUV when the GFP concentration was increased from *X* = 0 nM to *X* = 5.3 nM over a period of about 30 min. Initially, nanotubes (I, white arrow) protruding from the spherical mother vesicle into the vesicle interior were stabilized by negative spontaneous curvature arising from the solution asymmetry between a 300 mM sucrose solution inside the vesicle and a 150 mM NaCl + 40 mM sucrose solution outside the vesicle. The addition of GFP to the exterior solution generated a less negative spontaneous curvature that transformed the in-tubes into in-buds (II) and eventually into out-buds (III, IV). The response of another GUV exposed to the same time-dependent GFP concentration is displayed in Supplementary Fig. 9. **b** Time-dependent shape evolution of a single GUV exposed to the constant GFP concentration *X* = 7.8 nM. At time *t* = 0 s, the GUV exhibits a short outward-pointing necklace consisting of two small spheres (white circles) that were successively released from the GUV via neck fission. The neck between the two small spheres was cleaved after about 19 s, the neck between the remaining bud and the mother vesicle after about 117 s. **c** Classification of stable GUV morphologies into three fractions as observed in two independent experiments: for each GFP concentration, we determined the fraction of shapes with inward-pointing membrane protrusions and total spontaneous curvature *m* < 0, prolate shapes with no visible protrusions and *m* ≃ 0, shapes with outward-pointing protrusions and *m* > 0 as illustrated by the three images on the right. All data displayed in the present figure were obtained for 1 mol% anchor-lipids. All scale bars: 5 μm. Source data (number of vesicles per condition) for panel **c** are provided in the Source Data file.

To obtain GUVs with such reservoirs for membrane area, we prepared GUVs that contained 1 mol% anchor-lipids for GFP binding. Initially, we exposed these GUVs to asymmetric salt–sugar solutions without GPF to generate a large *negative* spontaneous curvature *m*_ini_ ≃ −8.8 μm^−1^ of the membranes as in a previous study^18^. Osmotic deflation then produced spherical mother vesicles that were connected to inward-pointing nanotubes (see panel I of Fig. 5a). The tubes were only visible because of the red membrane dye but had a width below optical resolution. We then added GFP to the exterior solution. As before, the GFP became bound to the anchor-lipids in the outer leaflet of the GUVs, thereby generating a *positive* contribution, *m*_GFP_ > 0, which leads to the total spontaneous curvature *m* = *m*_ini_ + *m*_GFP_. Increasing the GFP concentration, we observed a sequence of GUV morphologies as displayed in Fig. 5a. Furthermore, when the total spontaneous curvature *m* = *m*_ini_ + *m*_GFP_ exceeded the threshold value m_*_ ≃ 5.8 μm^−1^ as estimated above, the closed membrane necks of the out-buds again underwent fission and the buds were cleaved from the mother vesicle as shown in Fig. 5b.

Finally, we studied whole populations of GUVs with an initial spontaneous curvature *m*_ini_ ≃ −8.8 μm^−1^, again generated by asymmetric salt–sugar solutions. We then exposed these vesicles, which contained 1 mol% anchor-lipids, to different GFP concentrations and sorted the observed GUV morphologies, for each GFP concentration *X*, into three fractions (Fig. 5c). For GFP concentrations between 3.1 and 4.7 nM, which generated  a  contribution *m*_GFP_ between 5.8 and 8.8 μm^−1^ to the total spontaneous curvature, the majority of GUVs lost inward-pointing protrusions implying that the total spontaneous curvature was close to zero and that the initial spontaneous curvature *m*_ini_ =  −*m*_GFP_ had a value between  −5.8 and  −8.8 μm^−1^ in reasonable agreement with the value *m*_ini_ ≃ −8.8 μm^−1^ obtained previously^18^. This agreement demonstrates that we can estimate even rather high spontaneous curvatures by using relatively simple sorting criteria for the observed vesicle morphologies. To obtain a more precise value for the GFP-generated spontaneous curvature *m*_GFP_, needed to compensate the initial spontaneous curvature *m*_ini_ from the salt–sugar asymmetry, we observed the complete shape evolution of single GUVs as a function of GFP concentration and determined the concentration value at which the vesicles lost their nanotubes and became prolate. Averaging over five such experiments, we obtained *m*_ini_ = (−8.3 ± 1.8) μm^−1^ (Supplementary Table 1).

## Discussion

In this study, we developed a synthetic membrane system for which we can vary the spontaneous curvature of the membranes in a controlled and quantitative manner. The system is based on GUVs and the reversible binding of proteins to the outer leaflets of the GUV membranes. We first identified a broad range of GFP solution concentrations for which the GFP coverage of the membranes increased linearly with the concentration (Supplementary Fig. 1 and “Methods” section, Eq. (3)). We then showed, based on the detailed comparison between experiment and theory, that the spontaneous curvature generated by the membrane-anchored GFP increases linearly with the GFP coverage as in Eq. (1) and Fig. 2a. The GFP-generated curvature is surprisingly large and comparable with the curvature generated by membrane-bound BAR-domain protein such as amphiphysin. As we increased the GFP coverage, prolate GUV shapes were transformed into dumbbell shapes, consisting of two spherical membrane segments connected by a closed membrane neck (Figs. 1c, 2b–d, and 3). The relative size of the two spherical sub-compartments was controlled by the volume-to-area ratio *v* of the prolate GUV (Fig. 2c and Table 1).

The volume-to-area ratio can be varied in a controlled and quantitative manner using two different and complementary methods. First, this ratio can be controlled by changing the vesicle volume via the osmotic conditions. This first method was used to obtain the shape transformations displayed in Figs. 2–4. Second, the volume-to-area ratio of the mother vesicle can be changed by coupling this vesicle to a reservoir of membrane area. The latter method has been used for the GUVs in Fig. 5, where the area reservoir was provided by membrane nanotubes.

Once a dumbbell shape with a closed membrane neck has been formed, this morphology persists as we further increase the GFP concentration and, thus, the spontaneous curvature for fixed volume-to-area ratio. However, for sufficiently large GFP concentrations, the closed membrane neck undergoes fission and the GUV is divided into two daughter vesicles (Figs. 3, 4, and Supplementary Fig. 6). These experimental observations confirm recent theoretical predictions that the spontaneous curvature *m* generates a constriction force *f* as described by Eq. (2). For the lipid and protein compositions studied here, closed membrane necks underwent fission when they experienced such a constriction force *f* above 26 pN (Table 1).

The synthetic membrane system introduced here provides a versatile method to precisely control the spontaneous curvature which will allow us, in future studies, to investigate unexplored regions of the morphology diagram (Fig. 2c), including the stability regimes for multi-sphere shapes with more than two spheres. In the present study, we used His-tagged GFP bound to anchor-lipids in order to generate membrane curvature and fission. However, curvature-driven fission provides a *generic* fission mechanism that can be explored using other membrane-bound proteins. One example is provided by His-tagged iLid bound to the same anchor lipids (see Supplementary Fig. 10 and Supplementary Table 2). The latter figure demonstrates that comparable solution concentrations of His-tagged iLid and His-tagged GFP generate similar values of the spontaneous curvature when the two types of proteins are bound to GUV membranes with the same lipid composition and only 0.1 mol% NTA anchor-lipids. iLid belongs to a particularly interesting class of proteins, which are able to form photo-switchable protein dimers^19^. Such dimers should lead to an increased spatio-temporal control over the process of neck fission and vesicle division.

In the present study, we controlled the spontaneous curvature by binding proteins, which were dissolved in the exterior solution, to anchor-lipids in the outer leaflet of the GUV membranes. If we dissolve these proteins in the interior solution and bind them to the anchor lipids in the inner leaflet, we generate a negative spontaneous curvature that favors the formation of inward-pointing buds.^14^ The subsequent fission of the closed membrane neck between the mother vesicle and the in-bud will then lead to the formation of a small vesicle within the mother vesicle, a membrane remodeling process that proceeds in close analogy to endocytosis of cellular membranes. On the other hand, a positive spontaneous curvature that favors the formation of out-buds as in Figs. 2 and 3 can also be generated by solutes within the interior solution when these solutes form a depletion layer adjacent to the inner leaflet of the GUV membrane^20^.

It is instructive to compare our method for GUV division as introduced here with other methods that have been developed for the same purpose. One alternative method that has been pursued for some time is based on the reconstitution of the bacterial division machinery provided by FtsZ proteins. The formation of relatively large rings of these proteins within GUVs has been observed, albeit with rather low frequency^1^. For about 1.2% of the GUVs, Z rings were observed to induce progressive constrictions of the GUVs, in some cases leading to two subcompartments connected by closed membrane necks. However, the subsequent separation of these two subcompartments as observed in our study (Fig. 3, Supplementary Movies 2 and  3) has not been reported in ref. ^1^. Furthermore, the buffer used in this latter approach involved two nucleotides, ATP and GTP. In the absence of GTP, no progressive constrictions of GUVs have been observed.

In ref. ^2^, extruded vesicles with a diameter of 200 nm were exposed to His-tagged GFP. The vesicle membranes contained 20 mol% NTA anchor-lipids and were exposed to a GFP solution concentration of 5 and 20 μm. As a consequence, the anchor-lipid mole fraction was at least 20 times higher and the GFP solution concentration was at least 128 times larger than in our systems. In fact, it was concluded in ref. ^2^ that crowding of membrane-bound GFP is a prerequisite for successful vesicle division. In contrast, our results clearly demonstrate, both for GUVs (Fig. 3, Supplementary Movies 2 and 3) and for extruded vesicles with a diameter of 200 nm (Fig. 4c) that vesicle division can be achieved in the dilute regime, in which the separation of the membrane-bound GFPs is much larger than their lateral size.

In ref. ^3^, a microfluidic-based method was used to mechanically split GUVs into two daughter vesicles. The GUVs were formed from double-emulsion droplets and subsequently flowed against the sharp edge of a wedge-shaped microfluidic junction. The resulting division process competed primarily with two alternative outcomes, bursting of the GUV and futile division attempts with no splitting (called ‘snaking’ in ref. ^3^). As a consequence, the probability for division was observed to depend strongly on the size of the GUV and to follow a bell-shaped curve with a maximum at a GUV diameter of about 6 μm. For the latter size, the division probability was about 0.38. Both for smaller and for larger sizes, the division probability rapidly decreased to zero: For smaller sizes, futile division attempts became the typical outcomes whereas larger GUVs were destroyed by bursting. For our division method, we have not yet studied the dependence on GUV size in a systematic manner but our experiments do not provide any evidence for such a dependence. Furthermore, the curvature-induced constriction force as given by Eq. (2) and displayed in Fig. 4a does not involve the GUV size which implies that our division method should be size-independent.

Our protocol for vesicle division does not require any coupling of the membranes to filaments or nucleotide hydrolysis. Thus, in contrast to the complex protein machinery that drives neck fission in vivo, our synthetic system for curvature-driven fission is chemically quite simple and involves only lipid membranes and one species of His-tagged proteins. Furthermore, the low density of the membrane-bound GFP leaves ample space for other proteins to be accommodated on or in the GUV membranes. Membrane proteins that could be added include ion channels and ion pumps that span the whole bilayer membranes. Therefore, the membrane-protein systems introduced here provide promising and extendible modules for the bottom-up approach to synthetic biology^21–23^.

## Methods

### Lipids and GUV production

Chloroform stock solutions of 1-palmitoyl-2-oleoyl-sn-glycero-3-phosphocholine (POPC) and 1-palmitoyl-2-oleoyl-sn-glycero-3-phospho-(1’-rac-glycerol) (sodium salt) (POPG) and cholesterol were mixed at a molar ratio of 7/1/2 with a final lipid concentration of 4 mM. As indicated in the main text 0.1 or 1 mol% of 1,2-dioleoyl-sn-glycero-3-[(N-(5-amino-1-carboxypentyl)iminodiacetic acid)succinyl] (nickel salt) (DGS-NTA) was added to the lipid solution. All lipids were obtained from Avanti Polar Lipids. If not indicated otherwise, membrane fluorescent dye DiD (1,1’-Dioctadecyl-3,3,3’,3’-Tetramethylindodicarbocyanine, 4-Chlorobenzenesulfonate Salt, Thermo Fisher Scientific) was added at 0.1 mol%. For most experiments, GUVs were grown on a polyvinyl alcohol (PVA) film^24^. For PVA film formation, glass slides were cleaned by rinsing in ethanol and double-distilled water. Subsequently, 50 μL of a 50 g/L  PVA (Merck) solution in water was spread on the glass slide to form a thin film. The PVA film was dried at 50 ^∘^C for 30 min. Then, 5 μL of 2 mM lipid solution in chloroform was spread on the PVA film and the solvent was evaporated in vacuum for 1.5–2 h. The lipid film was then pre-hydrated in a stream of water saturated nitrogen gas for 3 min. A chamber was build from the PVA and lipid-coated coverglass and a Teflon spacer, giving a total volume of about 700 μL. GUVs were then grown in 50 mM NaCl, 5 mM sucrose, and 5 mM TRIS pH 7.3 buffer. All chemicals were obtained from Sigma. GUVs produced in this way encapsulate a certain fraction of the hydrogel substrate (PVA), which leads to a refractive index difference between the interior and outside solution and to sedimentation of the GUVs to the bottom of the observation chamber. To compensate for the small solution asymmetry by encapsulating PVA from the swelling procedure, in some experiments an addtional 2 mM sucrose was added to the outer solution. This procedure increased the fraction of GUV with initially zero spontaneous curvature. To check the vesicle production procedure just described, another set of experiments was performed using electroformed GUVs. Here, 5 μL of 0.2 mM lipid solution in chloroform was speared on two 2 cm long platinum wires (wire to wire distance was 5 mm). Chloroform was evaporated in vacuum for 1.5–2 h and lipid films were hydrated in the same buffer as specified above. The electroformation voltage was increased stepwise to 3 V peak-to-peak and electroformation was performed at 50 °C.^25,26^. Experiments on electroformed GUVs led to identical results as those on GUVs grown on PVA films. However, electroformed GUVs in hight salt conditions often encapsulated smaller GUVs, which made PVA-grown GUVs preferable for the experiments.

### Coverage–concentration relationship for GFP

The dependence of the GFP coverage Γ on the GFP solution concentration *X* is displayed in Supplementary Fig. 1. The experimental data in this figure are well fitted by the linear relationship

3 $$\Gamma =\frac{69}{\upmu{{\rm{m}}}^{2}}\ \frac{X}{{\rm{nM}}}\quad {\rm{for}}\ 1\ {\rm{mol}} \% \ {\rm{anchor}}{\hbox{-}}{\rm{lipids}}$$

over the whole concentration range 0 < *X* ≤ 23.4 nM. The GFP concentration *X* = 23.4 nM leads to the GFP coverage Γ = 1615 μm^−2^ and an average GFP–GFP separation of 25 nm. For 0.1 mol% anchor-lipids, the prefactor 69 in Eq. (3) is reduced to 6.9. The largest GFP concentration to which we exposed the GUVs with 0.1 mol% anchor-lipids was *X* = 39 nM which led to the GFP coverage Γ = 269 μm^−2^, corresponding to an average separation of 61 nm between the membrane-bound GFPs.

### Curvature and stability of closed membrane neck

The dumbbell shapes displayed in Figs. 1c, 2c, d, and 3 consist of two spherical membrane segments connected by closed membrane necks. For such a dumbbell shape, the curvature radii *R*_1_ and *R*_2_ of the two spherical segments adjacent to the neck define the neck curvature^14^

4 $${M}_{{\rm{ne}}}\equiv \frac{1}{2}\left(\frac{1}{{R}_{1}}+\frac{1}{{R}_{2}}\right)\ ,$$

which can be directly obtained from the optical images of the GUV as in Fig. 1c. The closed neck is unstable and opens up if the neck curvature *M*_ne_ exceeds the spontaneous curvature *m*. Therefore, the membrane neck is stably closed if

5 $$m\ge {M}_{{\rm{ne}}}\quad ({\rm{stability}}\ {\rm{of}}\ {\rm{closed}}\ {\rm{neck}})$$

For a given vesicle batch with a certain spontaneous curvature, all dumbbell shapes must have a neck curvature that does not exceed the spontaneous curvature. As a consequence, the largest neck curvature provides the best estimate for the spontaneous curvature *m*. In general, the vesicles examined for a given batch differed in their area and volume and, thus, in their volume-to-area ratio *v*. Furthermore, in the stability regime for the dumbbell shapes, the rescaled neck curvature *M*_ne_*R*_ve_ depends only on *v* but not on the rescaled spontaneous curvature^14^, and the distribution of the neck curvature *M*_ne_ thus reflects the variations in membrane area and vesicle volume.

### Details of morphology diagram

For a vesicle of volume *V* and membrane area *A*, we use the vesicle size ${R}_{{\rm{ve}}}=\sqrt{A/(4\pi )}$ as the basic length scale. The vesicle morphologies then depend only on two dimensionless shape parameters^13^, the volume-to-area ratio

6 $$v\equiv \frac{V}{\frac{4\pi }{3}{R}_{{\rm{ve}}}^{3}}=6\sqrt{\pi }\frac{V}{{A}^{3/2}}\quad {\rm{with}}\,\,0 \;< \,v\, \le \,1$$

and the rescaled spontaneous curvature *mR*_ve_ as used in Fig. 2c. For symmetric bilayers with vanishing spontaneous curvature, the curvature model predicts that the shapes of minimal bending energy are prolates for 0.65 < *v* < 1, in agreement with the shapes A–C in Fig. 2c, d whereas metastable tube-like prolates are predicted for *v* < 0.65. Such shapes have also been observed in our experiments (see Supplementary Fig. 11). The latter observations are in accordance with the results of Monte Carlo simulations^27^.

Dumbbell shapes with a closed membrane neck are found in a certain subregion of the morphology diagram which is located between the two lines of limit shapes, *L*_1+1_ and *L*_2*_, that separate vesicle shapes with open from those with closed membrane necks (see Fig. 2c). Using the abbreviation $\overline{m}\equiv m{R}_{{\rm{ve}}}$, the line *L*_1+1_ has the functional form ref. ^13^

7 $$v=-\frac{1}{4{\bar{m}}^{3}}+\left(1-\frac{1}{2{\bar{m}}^{2}}\right)\sqrt{1+\frac{1}{4{\overline{m}}^{2}}}\quad {\rm{for}}\quad \bar{m}\ge \sqrt{2}$$

and the line *L*_2*_ is located at^14^

8 $$v=1/\sqrt{2}\quad {\rm{and}}\quad \bar{m}\ge \sqrt{2}\ .$$

Therefore, the two lines meet in the corner point with $\bar{m}=\sqrt{2}$ and $v=1/\sqrt{2}$ (see Fig. 2c).

### Constriction force around closed membrane neck

To derive the constriction force generated by the spontaneous curvature around a closed membrane neck, we consider a convenient parametrization of a dumbbell shape, consisting of two hemispheres connected by two unduloid segments that form a narrow neck of neck radius *R*_ne_^14,28^. The dumbbell with a closed neck is obtained in the limit of zero *R*_ne_. To reveal the curvature-induced constriction force *f*, we first consider an external constriction force *f*_ex_ compressing the neck. In such a situation, the bending energy *E*_be_ of the dumbbell has the form

9 $${E}_{{\rm{be}}}({R}_{{\rm{ne}}})\approx {E}_{{\rm{be}}}(0)+{f}_{{\rm{ex}}}{R}_{{\rm{ne}}}+8\pi \kappa (m-{M}_{{\rm{ne}}}){R}_{{\rm{ne}}}$$

up to first order in *R*_ne_. The closed neck is stable if the term proportional to the neck radius *R*_ne_ increases with increasing *R*_ne_ which implies

10 $${f}_{{\rm{ex}}}+8\pi \kappa (m-{M}_{{\rm{ne}}}) \, > \, \, 0\quad ({\rm{stably}}\ {\rm{closed}}\ {\rm{neck}}).$$

In the absence of an external force, that is, for *f*_ex_ = 0, we then obtain the curvature-induced constriction force *f* = 8*πκ*(*m* − *M*_ne_) as in Eq. (2). This constriction force is proportional to the curvature difference *m* − *M*_ne_, which vanishes along the line *L*_1+1_. Once we have crossed the line *L*_1+1_ towards higher values of the shape parameter *mR*_ve_, the curvature difference *m* − *M*_ne_ increases monotonically as we increase the spontaneous curvature *m* for fixed volume-to-area ratio *v*. Indeed, for constant *v*, the neck curvature *M*_ne_ is determined by the two-sphere geometry of the dumbbell-shaped vesicle which remains unchanged as we increase *m* for constant *v*. For the dumbbell shapes displayed in Figs. 2c, d and 3, the numerical values of the spontaneous curvature *m*, the curvature difference *m* − *M*_ne_, and the constriction force *f* are displayed in Table 1.

### Transition state and free energy barrier for neck fission

The process of neck fission and vesicle division is schematically shown in Fig. 4b. This process represents a topological transformation from the one-vesicle state provided by the dumbbell shape to the two-vesicle state of two separate daughter vesicles. Both states have essentially the same bending energy because the closed neck of the dumbbell does not contribute to this energy. However, the two states have different topologies which implies that the Gaussian curvature term^16^ makes a different contribution to the one-vesicle and to the two-vesicle state. The latter contribution is equal to 4*πκ*_G_ for the one-vesicle state and to 8*πκ*_G_ for the two-vesicle state, where *κ*_G_ is the Gaussian curvature modulus. Therefore, the difference in free energy, *G*_2_ − *G*_1_, between the two-vesicle and the one-vesicle state is equal to 4*πκ*_G_. Both experimental studies^29,30^ and computer simulations^31^ indicate that the Gaussian curvature modulus is negative with *κ*_G_ ≃ −*κ*. For the lipid membranes studied here, we then obtain the estimate *κ*_G_ ≃ −48*k*_B_*T* which leads to the free energy difference *G*_2_ − *G*_1_ ≃ −603*k*_B_*T*. Therefore, neck fission and GUV division is a strongly exergonic process and can, in principle, occur spontaneously. However, the rate with which this process proceeds is governed by the free energy barrier that separates the one-vesicle from the two-vesicle state.

The associated transition state that creates this free energy barrier corresponds to the intermediate state in Fig. 4b. In order to cleave the neck, we have to create two bilayer pores with a diameter that is comparable to the size of the closed membrane neck before fission. The resulting free energy barrier is governed by the edges of these two pores and the associated edge energy, which is equal to the edge tension *λ* times the combined circumference of the two pores. To lower the barrier by a significant amount, the constriction force generated by the spontaneous curvature must perform mechanical work that is comparable to this edge energy^14^. One then finds that the neck undergoes fission if the spontaneous curvature exceeds the threshold value m_*_ ≡ *λ*∕(2*κ*). For a vesicle membrane with 0.1 mol% anchor-lipids and a bending rigidity *κ* ≃ 48*k*_B_*T*, the threshold value X_*_ ≃ 31 nM for the GFP concentration (Supplementary Fig. 6) implies a threshold value m_*_ ≃ 5.8 μm^−1^ for the spontaneous curvature and an edge tension of about 2.3 pN, which belongs to the range of *λ*-values that has been obtained experimentally for lipid bilayers^32^.

### Bending rigidity measurements by fluctuation analysis

Membrane bending rigidity was measured by fluctuation analysis of the thermally induced motion of the membrane, based on the Fourier spectrum of the cross-sectional contour as obtained from discrete time series of optical snapshots^33^. Experiments were performed on an Axio Observer D1 microscope (Zeiss, Germany) with a 40 × 0.6 air lens in phase contrast mode. Imaging was performed using a low noise liquid-cooled digital camera pco.edge 5.5. We acquired a total of 2000–4000 snapshots per vesicle with an exposure time of 200 μs. Only vesicles with optically resolvable fluctuations and no visible defects were considered for analysis. The bending rigidity of the ternary lipid membranes in the absence of GFP was measured to be *κ* = (48 ± 4)*k*_B_*T* at room temperature.

### Experiments using microfluidic chips

**Fig. 6:**
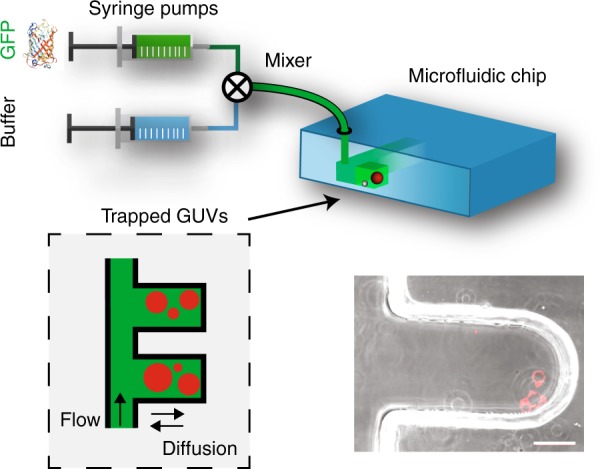
Design of microfluidic chips for the exposure of GUVs to different solution conditions. Cartoon and image of GUVs (red) located in microfluidic dead-end channels that acted as vesicle traps (bottom) and solution conditions outside the GUVs were set by computer-controlled syringe pumps (top). The solutes reached the GUVs by diffusion into the traps (Supplementary Movie 4). In this way, the GUVs were screened from mechanical perturbations arising from hydrodynamic flows in the main channel. Scale bar for the lower right image: 50 μm.

Experiments were conducted using polydimethylsiloxane (PDMS) microfluidic chips. The chip design consisted of a main channel with several dozen dead-end side channels (or cavities) of 150 μm width and length between 250 and 500 μm (see Fig. 6). By centrifugation at 50 × *g* for 5 min, GUVs were loaded into the microfluidic dead-end channels. The solution conditions of the main channel were precisely controlled using computer-controlled syringe-pumps and off-chip mixing of the solutes. The dead-end channels exchanged the solutes via diffusion with the main channel. Timescales for fluid exchange by diffusion were about 10 min (see Supplementary Movie 4), and GUVs were typically left to equilibrate for at least 15 min. The flow speeds in the main channel were 1–10 μL/min. This setup is useful for the investigation of GUV morphologies, because the GUVs are screened from the hydrodynamic flows in the main channel and do not experience hydrodynamic perturbations as in other microfluidic trapping methods^34^. Indeed, the flows are expected to decay on a length scale that is set by the dead-end channel width which was smaller than the channel length (Fig. 6). Because the GUVs still undergo thermal motion, they will eventually escape from the dead-end channel. However on the typical timescales of the experiments, the deeply trapped GUVs did not exhibit any significant motion (Supplementary Movie 4). To avoid membrane stresses or other undesired effects, osmotic conditions were only changed in small (10%) steps with sufficient equilibration time in-between steps. In this way, the solution osmolarity was increased up to 100 mM NaCl, 10 mM sucrose and 10 mM TRIS pH 7.35 buffer.

### GUV imaging and image quantification

Fluorescent images were obtained on a Leica SP5 or SP8 confocal microscope in transmission mode (40 × 0.75 or 40 × 0.6 air lens). Membrane dye DiD was excited using the 622 line (solid state laser) and GFP using the 488 line (argon laser). Emission was collected at 493–550 and 627–700 nm, respectively. To visualize membrane morphologies, images were contrast adjusted in the DiD channel.

GFP membrane coverage was quantified from confocal images obtained with a 40 × 1.3 oil immersion lens. To prevent bleed-through between the fluorescent signals, the membrane dye DiD was not present in these experiments. First, GUVs were prepared with 1 mol% DGS-NTA at 16 nM GFP solution concentration. Using imageJ^35^, the average fluorescence intensity along the vesicle contour was measured (Radial Profile Extended plugin, Philippe Carl). The fluorescent background level was estimated from GUV micrographs where DGS-NTA was absent, but GFP present in the solution at 16 nM. In this way, the solution GFP signal can be subtracted from the GFP membrane signal^36^. In separate experiments, GUV with 0.1 mol% Oregon green 488 fused to the lipid head group of DHPE lipid (Thermo Fisher Scientific) were prepared, which provided a reference signal of known membrane dye coverage. To compensate differences in the dyes, fluorescent intensities of water solvated GFP was compared to water solvated Oregon green 488 dye on the same confocal setup. Finally, by taking the area per lipid to be 0.55 nm^2^ ^37^, a conversion factor was obtained, which was then used to convert membrane fluorescent intensities to GFP surface coverage.

### GFP purification and expression

His-GFP (N-terminally His6-tagged green fluorescent protein, Addgene plasmid # 29663) was recombinantly expressed in *E. coli* and purified over a Ni2+−NTA column using standard protocols. Initial GFP concentration was measured using NanoDrop UV–Vis spectrometer to be 78 μM. Before experiments GFP was diluted in the GUV buffer at the desired concentration.

### STED experiments

STED microscopy was performed on a Abberior Instruments microscope with dye excitation at 640 nm and STED depletion at 775 nm using a pulsed laser source. STED alignment was accomplished using gold beads, by adjusting the focus of the excitation beam into the center of the donut-shaped depletion laser. Corrections for mismatches between the scattering mode and the fluorescence mode were achieved using TetraSpeck beads of four colors. Crimson beads of 28 nm diameter were used to measure the resolution of STED which was found to be about 35 nm. For STED experiments vesicles were doped with 1 mol% ATTO 647N DOPE dye (Sigma).

### Neck stability measurements using LUVs

LUVs were prepared by hydration of a dried lipid film (POPC with 10 mol% POPG, 20 mol% cholesterol, and 1  mol% DGS-NTA). Same ionic conditions as in the GUVs experiments were used and initial lipid concentration was 400 μM. After five freeze–thraw cycles, the lipid suspension was extruded through a 200 nm polycarbonate filter (Avanti mini extruder). LUVs were diluted in 75 mM NaCl, 7.5 mM sucrose, and 7.5 mM TRIS pH 7.35 buffer to a final lipid concentration of 40 nM. Then LUVs were incubated at 23 °C for 30 min at the indicated GFP concentration. Liposome sizes were measured using dynamic light scattering (MALVEN Zetasizer Nano ZS). Autocorrelation curves were checked for satisfactory fit to the model and average LUV sizes were reported.

### Recruitment of area reservoirs

GUVs were prepared by the well-established electroformation method, in which an alternating current is used to swell individual lipid bilayers from electrodes (see, for example, ref. ^18^). The lipid membranes were composed of POPC:cholesterol 9:1 with 1 mol% DGS-NTA lipid and 0.1 mol% DiD membrane dye. The GUVs enclosed 300 mM sucrose (Sigma) solution buffered with 10 mM TRIS buffer at pH 7.35. The vesicles were trapped in the microfluidic chips as described (Fig. 6) and the outside solution was exchanged in small steps up to 150 mM sodium chloride, 40 mM sucrose, 10 mM TRIS buffer. Subsequently, the solution GFP concentration was adjusted at constant osmotic conditions as described in the main text.

### Reporting summary

Further information on research design is available in the Nature Research Reporting Summary linked to this article.

## Supplementary information

Supplementary Information

Supplementary Movie 1

Supplementary Movie 2

Supplementary Movie 3

Supplementary Movie 4

Description of Additional Supplementary Files

Reporting Summary

## Data Availability

The source data underlying Figs. 2a, c, 3c, 4a, c, d, and 5c as well as Supplementary Figs. 1, 5a, c are provided in a separate Excel file labeled ‘Source Data’.

## Source data

Source Data
