# Supplementary material for: Controlled division of cell-sized vesicles by low densities of membrane-bound proteins
Source: Nat Commun. 2020 Feb 14;11:905. doi: 10.1038/s41467-020-14696-0 (PMC7021675; doi:10.1038/s41467-020-14696-0)
Supplement: Supplementary file 1 — Supplementary Information [file 41467_2020_14696_MOESM1_ESM.pdf]

Supplementary Information:

Controlled Division of cell-sized vesicles by low densities of membrane-bound proteins

Steinkühler et al

Supplementary Figures

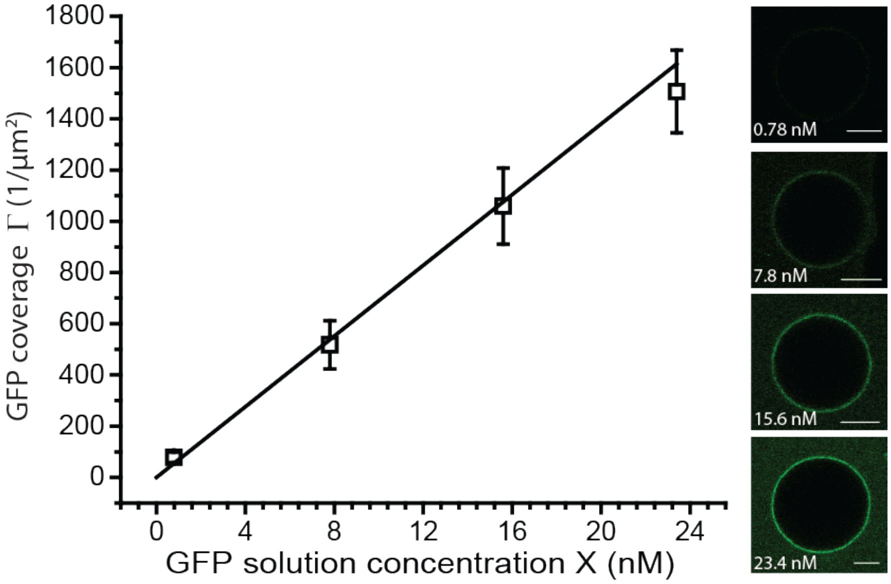

Supplementary Figure 1. (Left) Relation between solution GFP concentration *X* and membrane bound GFP density Γ. Each data point n=5 GUVs, errorbars show std. dev. The linear fit is $\Gamma = (69\ \pm 6)\ X$ µm^{-2}nM^{-1} with R^2 adj. 9.98 for 1mol% DGS-NTA GUVs, $\pm$ indicates the standard error of the fit. Replicate experiments (n=2) give the same slope within standard error. In experiments with 0.1mol% DGS-NTA the prefactor 6.9 is replaced with 69. Source data for the plot are provided in the Source Data file. (Right) Representative GUV Images for different solution concentrations. Note that at *X* = 0.78 nM the image is not completely black, but the GFP membrane signal is above the noise level when determined from the raw images.

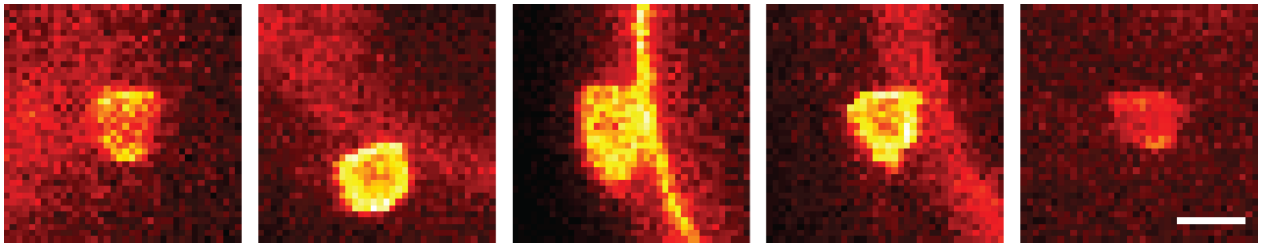

Supplementary Figure 2. Several STED images of a single bud diffusing along the membrane of the mother vesicle. The neck curvature is calculated from the radii of bud and mother vesicle, see equation (4) in the *Methods*. Fitting the bud contours by circles, we obtain the estimate 900 nm ± 70 nm (std. dev., n=5, raw images shown above) for the radius of the bud. The corresponding standard deviation for a neck curvature of 4 μm^{-1} is then given by $3\sigma \approx 0.4\ \mu m^{-1}$ as calculated by standard error propagation. The STED images were taken for solution concentration X = 15.6 nM and 0.1 mol% DGS-NTA. Scale bar 1 µm.

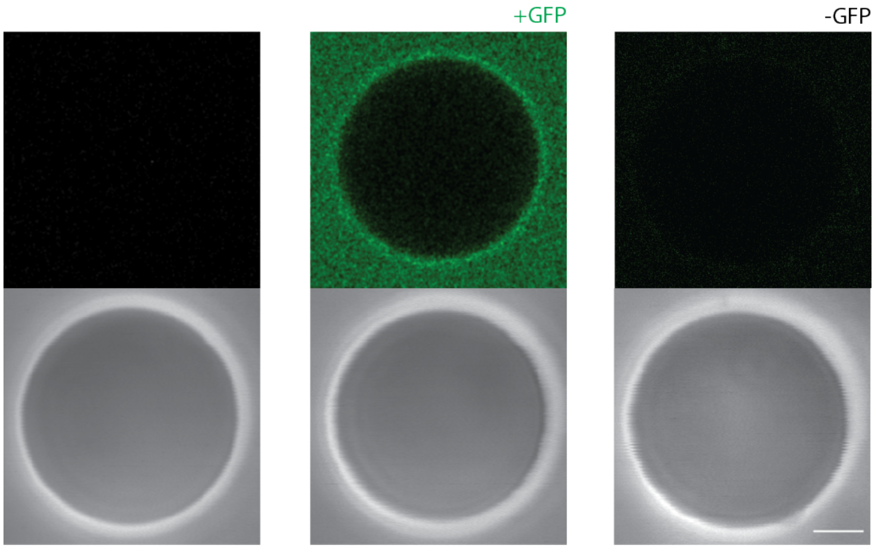

Supplementary Figure 3. Reversibility of GFP binding to DGS-NTA. Left-to-right: A single spherical GUV (1 mol% DGS-NTA) exposed to 3.9 nM GFP and subsequent washout with pure buffer (*X*=0 nM GFP). Between successive images, the system was equilibrated for 10 minutes. The equilibration time was sufficient to lower the GFP membrane coverage by more than 10-fold. Top row shows the GFP signal, the bottom row the phase contrast image of the GUV. In these experiments, the membrane contained no fluorescent dye to avoid the possibility of cross-talk between the fluorescence channels. Scale bar 5µm.

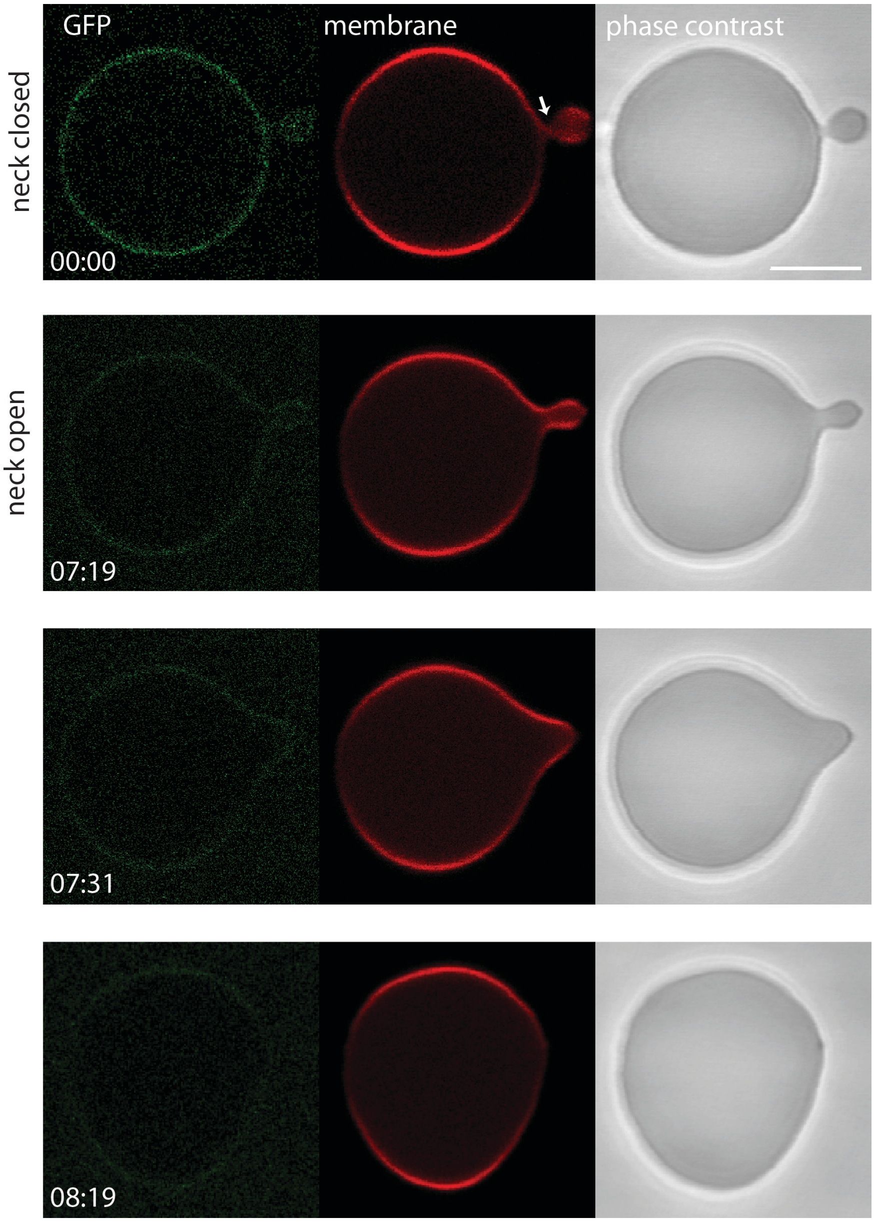

Supplementary Figure 4. Reversibility of shape transformations with GFP unbinding. Three channels are shown, GFP, membrane dye and phase contrast. Top-to-bottom: A single spherical GUV (0.1 mol% DGS-NTA, initial GFP coverage 7.8nM) has formed a closed and stable bud (arrow) by binding of GFP. GFP was washed away at constant osmotic conditions. The vesicle undergoes a series of shape changes that include opening of the neck, a transient pear-like shape and complete retraction of the bud. Time in min:sec and scale bar 5 µm. The complete time sequence is shown in Movie1.

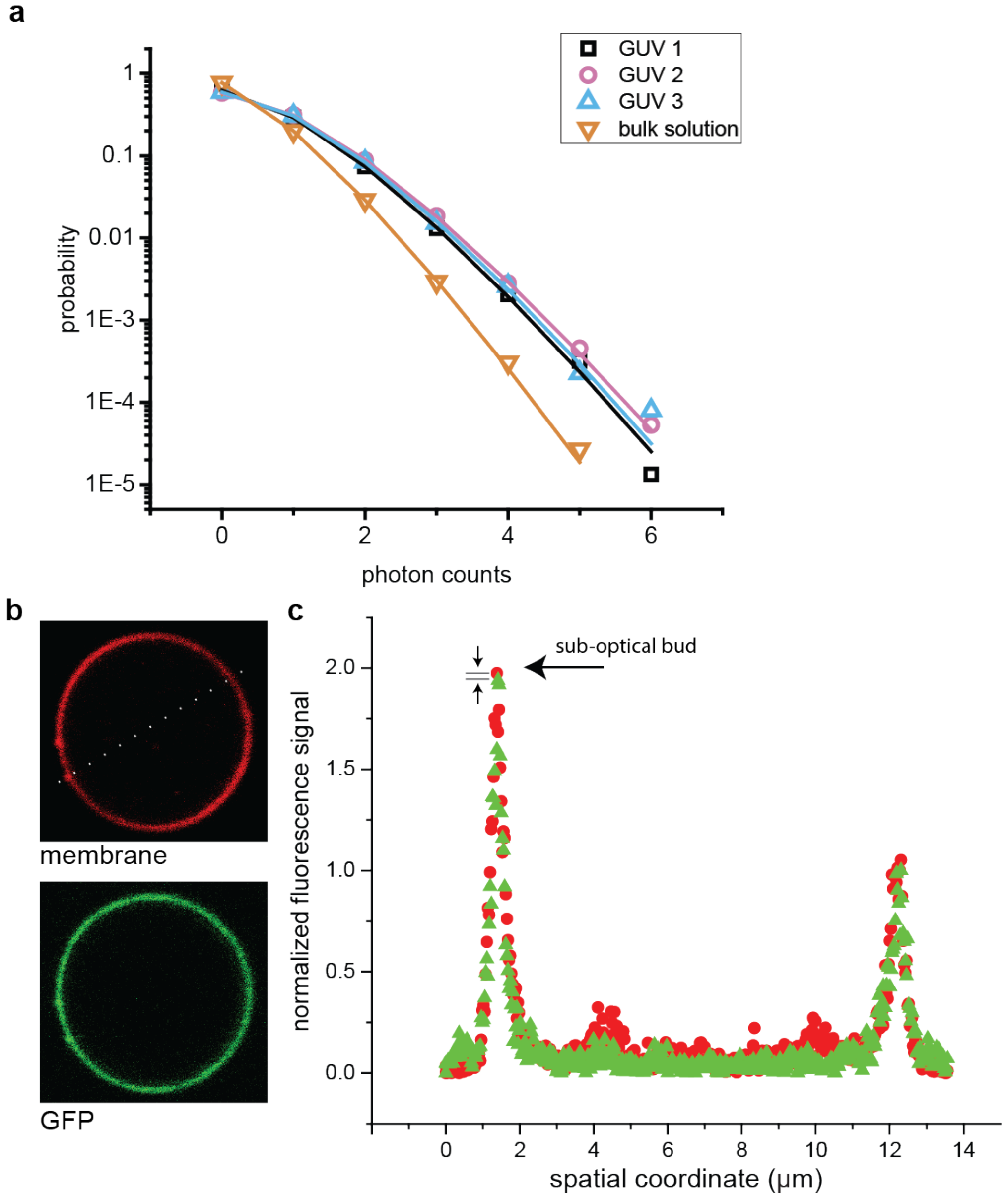

Supplementary Figure 5. (**a**) Photon counting histogram (PCH) at *X* = 7.8 nM solution concentration and 2 mol% DGS-NTA (see also Supplemental Methods below). The inverted triangles represent the histogram for the bulk solution, the other traces show measurements from equatorial cross sections of three GUV membranes. The photon distribution is a sensitive measure for the average number of fluorescent molecules *N* and for the brightness *ε* of each molecule [1]. Lines provide the best fits based on a globally fitted molecular brightness *ε* and using *N* as the only free parameter for each dataset ($\chi^2 \approx$ 1.05). The molecular brightness is proportional to the degree of GFP dimerization. The used GFP is optimized and known to be monomeric for the solution concentrations studied here. [2] Thus, the fit to a single value of molecular brightness *ε* for both free and bound GFP demonstrates that GFP remains a monomer when bound to the membrane. (**b**) Mother vesicle (DGS-NTA 2mol% and GFP solution concentration *X* = 7.8 nM) with two small buds observed by confocal microscopy. For these conditions,

the buds were below optical resolution and appeared as bright, diffraction-limited spots on the lower left part of the membrane contour. Membrane (red) and GFP (green) channels are shown separately. The diameter of the GUV is about 11 µm; and **(c)** Fluorescent signal, normalized to the flat membrane segment (upper right of the membrane contour), versus the spatial coordinate along the dashed white line in panel b. Because of the finite optical resolution, the fluorescent signal integrates the signal from the bud and neck region. We differentiate between the signal from the membrane dye I_{bud}^{DID} and GFP I_{bud}^{GFP}. The fluorescent signal from the flat membrane segment are denoted as I_0^{DID} and I_0^{GFP}. Equal density of GFP per membrane area in the bud/neck and flat membrane segment implies I_{bud}^{GFP} / I_{bud}^{DID}=I_0^{GFP} /I_0^{DID}. This is equivalent to $\Delta := \frac{I_{bud}^{GFP}}{I_0^{GFP}} - \frac{I_{bud}^{DID}}{I_0^{GFP}} = 0$. The measured Δ is the difference in red and green peak intensity (two short horizontal lines close to the left peak) of the normalized signals. Measurements on 4 different buds gave Δ≈ $[0.114, -0.315, 0.0356, 0.0488]$ which corresponds to an average of Δ ≈ - 0.04 +/- 0.2 (n=4, std. dev.) which indicates negligible enrichment or depletion of membrane-bound GFP in the curvature membrane segments of membrane neck and bud. Source data for panels **a** and **c** are provided in the Source Data file.

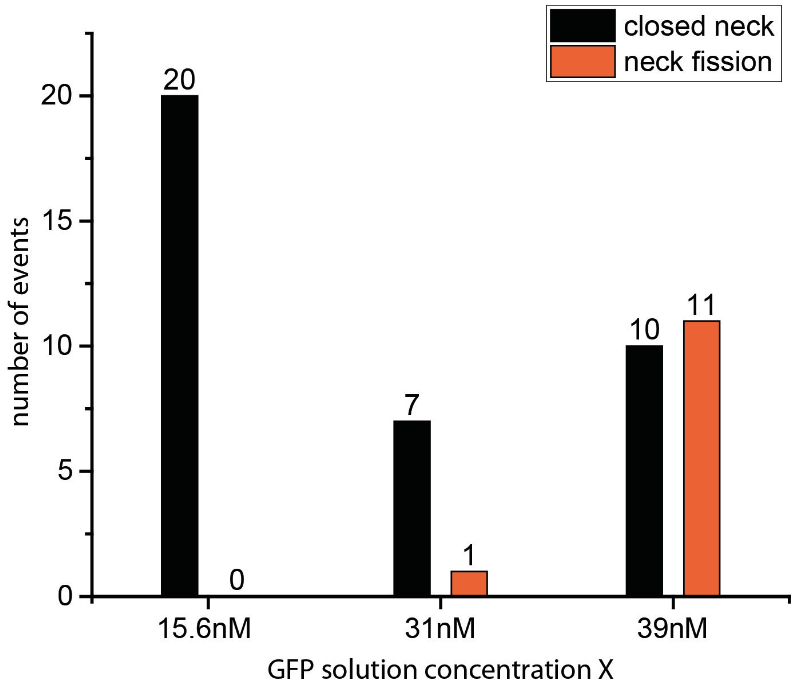

Supplementary Figure 6. Optical observation of neck stability from optical microscopy and bleaching experiments on single vesicles (GUVs) within one hour after GFP addition at the indicated concentration. DGS-NTA mole fraction was 0.1 mol%. Membrane spontaneous curvatures were approximately 2.9 μm^{-1}, 5.8 μm^{-1} and 7.3 μm^{-1}. Experiments were pooled from at least three independent experiments.

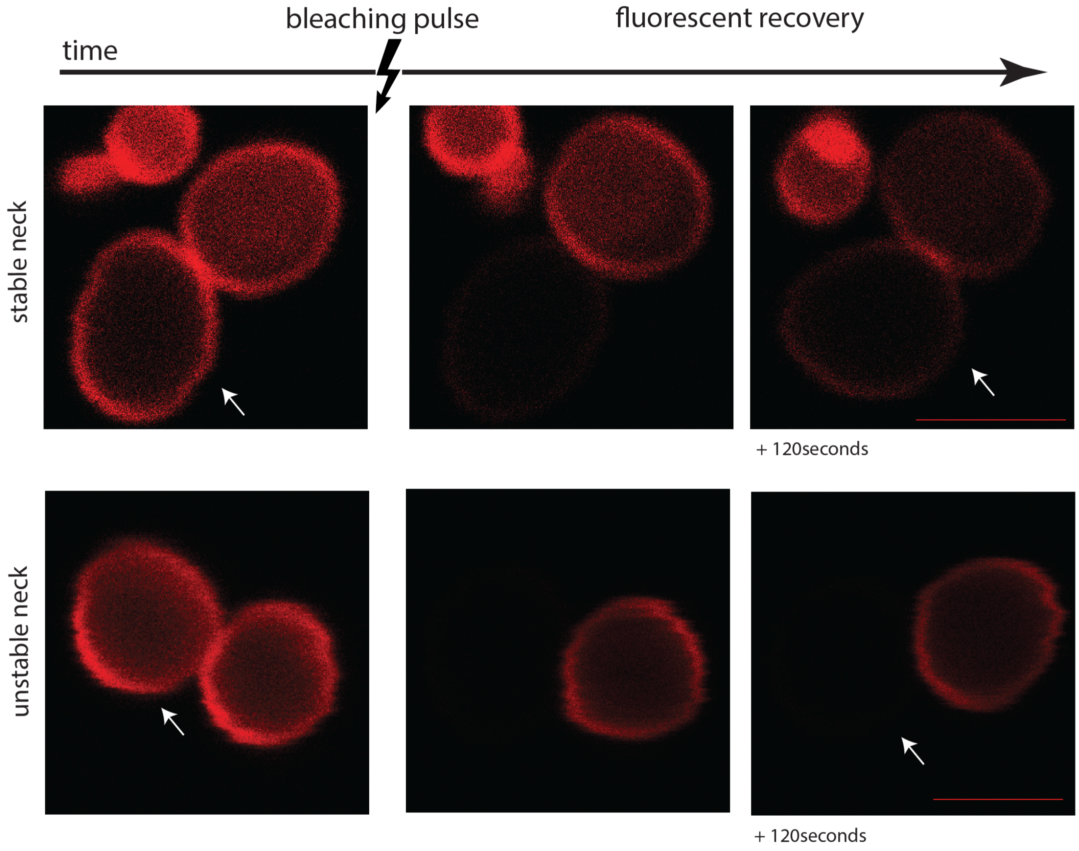

Supplementary Figure 7. Fluorescent bleaching assay to test membrane neck stability. The image in the top row were obtained for a spontaneous curvature of 1 μm^{-1}, the membrane dye (red) is distributed by diffusion across the closed and stable membrane neck. The images in the bottom row correspond to higher spontaneous curvature of 5.8 μm^{-1} and provide an example for neck fission, which was revealed by lack of fluorescent recovery on the bleached vesicle. Scale bars (red) 5 μm.

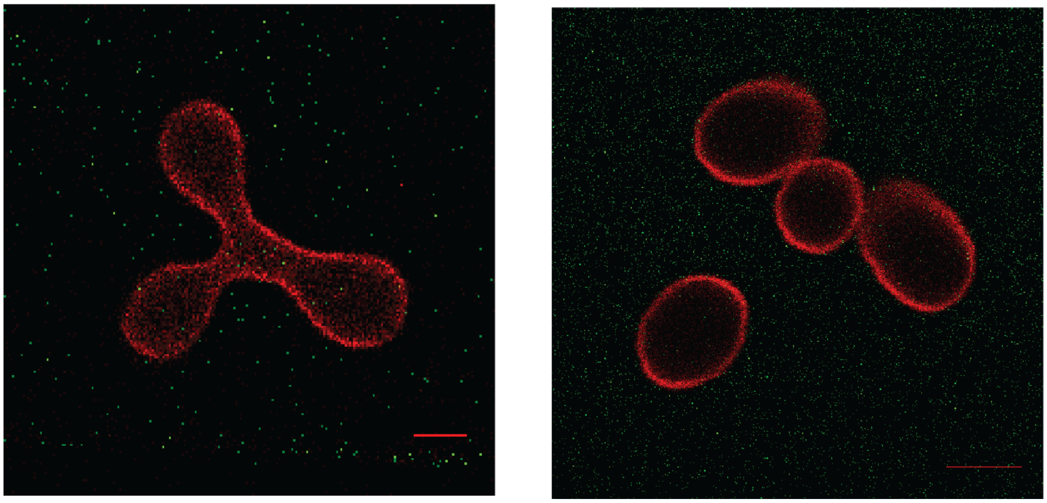

Supplementary Figure 8. Membrane fission of non-axisymmetric shapes after formation of closed necks at GFP concentration X=39 nM and DGS-NTA molar concentration of 0.1 mol%. Scale bar 5 µm.

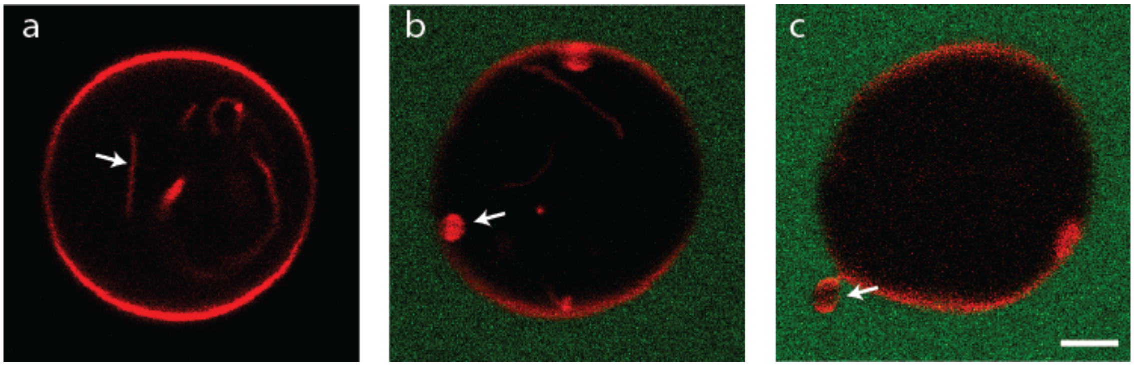

Supplementary Figure 9. Shape evolution of a another GUV when exposed to the same time-dependent GFP concentration as in Fig 5a: (a) Initially, the GUV forms inward-pointing nanotubes of sub-optical diameter which implies a large negative spontaneous curvature; (b) For larger GFP concentrations, necklace-like tubes and single buds are observed, corresponding to a less negative spontaneous curvature; and (c) For the final GFP concentration X = 5.3 nM , the GUV membrane forms an outward-pointing bud, which implies a positive spontaneous curvature. DGS-NTA 1mol%. Scale bar 5 μm.

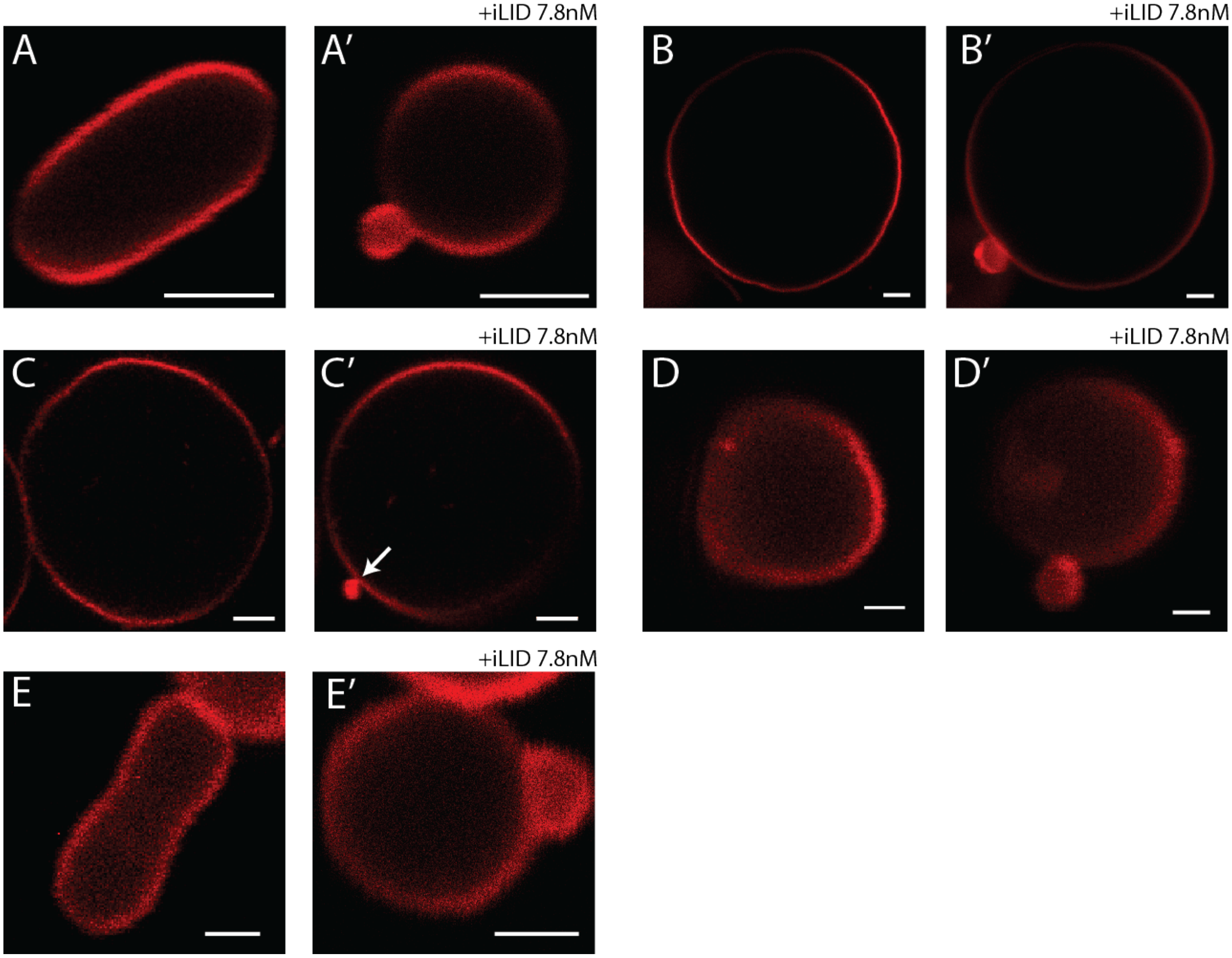

Supplementary Figure 10. Images of deflated GUVs (DGS-NTA 0.1mol%) in symmetric solution conditions (A,B,C,D,E) and exposed to 7.8 nM His-tagged iLiD in the outer solution (A',B',C',D',E') . GUVs shown were obtained from three independent repeat experiments. Scale bar indicates 5µm (A,A',B,B',C,C') or 2µm (D,D',E,E'). The parameter values for the budded GUVs are displayed in Supplementary Table 2.

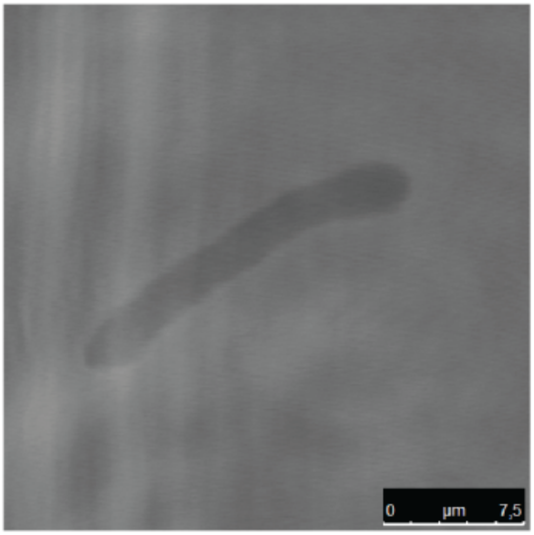

Supplementary Figure 11. Metastable prolate vesicle observed in the absence of GFP (X = 0 nM) for volume-to-area ratio v of approximately 0.51.

Supplementary Tables

GUV	GFP X [nM]	m_{ini} [1/µm]
1	3	-5.6
2	4.75	-8.8
3	3.9	-7.3
4	5.3	-9.9
5	5.3	-9.9

Supplementary Table 1. Single GUV study to estimate the GFP concentration X that leads to retraction of nanotubes stabilized by an initial spontaneous curvature m_{ini}. The mean value and S.D. is $m_{\text{ini}} \approx - 8.3 \pm 1.8\ \mu m^{-1}$.

Shape	**A'**	**B'**	**C'**	**D'**
R_{ve} [µm]	4.28	23.66	15.73	5.32
v	0.90	0.98	0.99	0.94
X_{iLID} [nM]	7.8	7.8	7.8	7.8
$m_{est}R_{ve}$	2.31	12.78	8.49	2.87
M_{ne} [µm^{-1}]	0.53	0.21	0.42	0.54
$m_{est} - M_{ne}$ [µm^{-1}]	0.01	0.33	0.12	0

Supplementary Table 2. Vesicle size R_{ve} estimated from the radii of buds and mother vesicles, volume-to-area ratio v, molar concentration X_{iLid} of His-tagged iLid protein, and rescaled spontaneous curvature $m_{est}R_{ve}$ for the four vesicle shapes A',B',C',D' depicted in Supplementary Fig. 10. The last two rows display the neck curvature M_{ne} as obtained from equation (4) in the main text and the curvature difference $m_{est} - M_{ne}$. The mole fraction of the DGS-NTA anchor-lipids was 0.1 mol%. The spontaneous curvature for the dilute iLID coverage at X_{iLID}=7.8 nM was estimated from the largest neck curvature of shape D' to be m_{est}=0.54 µm^{-1}. Comparison with equation (1) in the main text shows that this spontaneous curvature is of the same order of magnitude as the one generated by 7.8 nM His-tagged GFP. Shape E' in Supplementary Fig. 10 was not included in this analysis because of the deformation of the contact zone between bud and mother vesicle.

Supplementary Methods

PCH Histograms

PCH measurements were obtained using a 63x (1.2 NA) water immersion objective on a Leica SP5 setup. The sample was excited at 488 nm and fluorescence emission was collected in the band 500-550 nm using a filter cube. Photon counting was accomplished by avalanche photodiodes (Leica, Wetzlar, Germany) with a sampling frequency of 500Hz. PCH fitting was performed by ISS VistaFCS LE v3.6_70 software.

iLid expression and purification

pQE-80L iLID (C530M) and pQE-80L MBP-SspB Nano were gifts from Brian Kuhlman (Addgene plasmids #60408 and #60409 respectively). pQE-80L iLID (C530M) expresses iLID with an N-terminal His6-tag. All proteins were recombinantly expressed in E. coli following standard protocols. In short, 10 ml overnight cultures were inoculated into 1L LB medium with the appropriate antibiotic and grown at 37 °C shaking at 200 rpm till the OD_{600} = 0.6-0.8. The protein expression was induced with 500 µM IPTG and the cultures were grown overnight at 16°C. HIS6-iLID were then purified by Ni_{2+} affinity chromatography. Bud formation experiments with iLiD were performed identical to experiment with GFP.

Supplementary References

1. Müller, J.D., Y. Chen, and E. Gratton, *Resolving heterogeneity on the single molecular level with the photon-counting histogram.* Biophysical journal, 2000. **78**(1): p. 474-486.
2. Zacharias, D.A., et al., *Partitioning of lipid-modified monomeric GFPs into membrane microdomains of live cells.* Science, 2002. **296**(5569): p. 913-916.
