# Supplementary material for: Controlled division of cell-sized vesicles by low densities of membrane-bound proteins
Source: Nat Commun. 2020 Feb 14;11:905. doi: 10.1038/s41467-020-14696-0 (PMC7021675; doi:10.1038/s41467-020-14696-0)
Supplement: Supplementary file 6 — Description of Additional Supplementary Files [file 41467_2020_14696_MOESM6_ESM.pdf]

Title: Supplementary Movie 1
Description: Time evolution of the shape of a GUV that is exposed to a decreasing GFP concentration X in the exterior solution. The movie displays two channels, the GFP channel (green) on the left and the membrane channel (red) on the right. The GFP solution concentration is reduced from the initial value X = 7.8 nM at time 00:00 min:sec to X = 1.2 nM at time 06:51 min:sec. At the latter time, the membrane neck connecting the small and the large sphere starts to open up and the small sphere starts to become retracted into the large one. This retraction is completed after 07:36 min:sec. This time evolution directly demonstrates the reversible binding of the His-tagged GFP to the GUV membrane. At time 07:17 min:sec, the optical resolution is increased to obtain a better view of the membrane neck. The GUV membrane contained 0.1 mol% anchor lipids (DGS-NTA), the GFP was washed away at constant osmotic conditions. Some snapshots of this movie are displayed in Supplementary Fig. 4.

Title: Supplementary Movie 2
Description: Division of a dumbbell-shaped GUV, induced by an increase of the GFP concentration in the exterior solution from 0.78 to 15.6 nM. Additional GFP is added after 02:13 min:sec, neck fission occurs after 07:27 min:sec, and the two daughter vesicles are well separated after 07:41 min:sec. The GUV membrane contained 1 mol% anchor-lipids (DGS-NTA). Two snapshots of this movie are shown in Fig. 3d of the main text.

Title: Supplementary Movie 3
Description: Three dimensional reconstruction of GUV shape transformations based on confocal z-stacks. The movie shows a series of images for a prolate GUV (X = 0 nM) which forms a transient dumbbell-shape upon GFP adsorption and divides into two spheres at a final GFP concentration of X = 7.8 nM. The GUV membrane shown in red contained 1 mol% anchor-lipids (DGS-NTA).

Title: Supplementary Movie 4
Description: Washout of GFP (shown in green) from the microfluidic dead-end channel. GUVs were added in the main channel to visualize the flow profile. The dead-end channel effectively screens flow from the trapped GUV. Flow-speed in the main channel was 5 µL/min. Time is given in min:sec and the scale bar is 50 µm.
